# Two new species of *Limonia* Meigen, 1803 from Northwest China (Diptera, Limoniidae)

**DOI:** 10.3897/zookeys.971.35875

**Published:** 2020-09-24

**Authors:** Jinlong Ren, Ding Yang

**Affiliations:** 1 Key Laboratory of the Pest Monitoring and Safety Control on the Crop and Forest, College of Agronomy, Xinjiang Agricultural University, Urumqi 830052, China China Agricultural University Beijing China; 2 Department of Entomology, College of Plant Protection, China Agricultural University, Beijing 100193, China Xinjiang Agricultural University Urumqi China

**Keywords:** key, Limoniinae, Ningxia, Shaanxi, taxonomy

## Abstract

Two new species of *Limonia* Meigen, 1803, *Limonia medexocha***sp. nov.** and *Limonia subcosta***sp. nov.** are described and illustrated from Northwest China. The following five species are re-described and reported from China for the first time: *L. macrostigma* (Schummel, 1829), *L. phragmitidis* (Schrank, 1781), *L. stigma* (Meigen, 1818), *L. sylvicola* (Schummel, 1829) and *L. taurica* (Strobl, 1895). A key to adult males of *Limonia* from Northwest China is presented.

## Introduction

The genus *Limonia* Meigen, 1803 includes 181 known species worldwide, which are distributed in the Oriental (64 species), Holarctic (73 species), Afrotropical (13 species) and Australasian/Oceanian realms (36 species) (Oosterbroek 2019). Prior to this study 24 species were known from China. Adults of *Limonia* are moderately hydrophilic and mesophilous, often found in forest and open meadow biotopes (Savchenko 1985).

Northwest China includes the following six provinces: Xinjiang, Shaanxi, Ningxia, Gansu, Qinghai, and Inner Mongolia (western area). In this region the Altai Mountain (northern Xinjiang) and Qinling Mountain (southern Gansu and Shaanxi) are considered biodiversity hotspots in China (Liu and Chen 2014). However, no species of *Limonia* were known to occur in these mountains, and in this paper seven species, including two new species, are recorded. A key to adult males of *Limonia* from Northwest China is provided.

## Material and methods

The specimens were collected in northwestern China from July to August 2016–2017 using sweep netting, light trapping, and Malaise trapping. The specimens were studied and illustrated with a ZEISS Stemi 2000-c stereomicroscope; the photo illustrations were taken under a Canon Mark IV with Canon MP-E 65 mm lens. The descriptions were based on specimens preserved in 95% alcohol. Genitalic preparations of males were made by macerating the apical abdomen in cold 10% NaOH for 12–15 hours. After examination, preparations were transferred to fresh glycerin for preservation and stored in a microvial pinned below the specimen. The morphological terminology mainly follows McAlpine (1981). Type specimens are deposited in the Entomological Museum of China Agricultural University (**CAU**), Beijing, China.

## Taxonomy

### Limonia

Taxon classificationAnimaliaDipteraLimoniidae

Meigen, 1803

B1107697-ED30-5DA5-AA7F-B232B815030E

Limonia Meigen 1803: 262; Lackschewitz and Pagast 1940: 4–5; Geiger 1986: 106; Savchenko 1989: 328–330.

#### Type species.

*Tipula tripunctata* Fabricius, 1781 (subsequent designation by Westwood 1840) [= *phragmitidis* (Schrank, 1781)].

#### Remarks.

*Limonia* is characterized in the family by the following features: body color from yellow to gray, brown or black; medium-sized (body length 5.3–12.1 mm, wing length 5.4–13.2 mm); first thoracic segment elongate; episternum setose; tarsal claw with three to five teeth; wing wide with well-developed anal angle, pattern ranging from completely transparent or patternless to smoky or with dotted markings; Sc_1_ apically reaching from base of Rs to branching point of Rs; Sc_2_ close to apex of Sc_1_; terminal section of R_1_ continuing direction of R_1_ and longer than R_2_ (often at least two times longer than R_2_); discal cell closed; basal deflection of CuA_1_ at or slightly before branching point of M; male genitalia with wide ninth tergite slightly emarginate at posterior margin; gonocoxite with wide but often low ventromesal lobe; gonostylus single, situated apically, wider at base, narrowed and slightly arched at apex; aedeagus simple, elongate with bifid apex that is turned into the ventral margin; paramere wide at base; cercus of female terminalia slightly turned upwards (Savchenko 1985; Reusch and Oosterbroek 1997; Stary and Salmela 2004; Kolcsár et al. 2017; Podenas and Podeniene 2017; Starý 2018).

### A key to adult males of *Limonia* from Northwest China

**Table:** 

1	Wing with only one spot located at R_2_ (Figs 3, 12)	**2**
–	Wing with at least two spots located variously at basal Rs, apical Sc or R_2_ (Figs 19, 28, 37, 47, 57)	**3**
2	Body brown; wing with a distinct brown stigma (Figs 1, 3); occiput black brown without markings (Fig. 2); prescutum with three longitudinal stripes (Fig. 2); gonostylus with slender apex; paramere with slender and pointed apex (Figs 4–7)	***L. macrostigma* (Schummel, 1829)**
–	Body light yellow; wing with one indistinct pale brown stigma (Figs 10, 12); occiput yellow with Y-shaped marking (Fig. 11); prescutum with one longitudinal stripe (Fig. 11); gonostylus with obtuse apex (Figs 9, 10); paramere with short and obtuse apex (Figs 13–16)	***L. medexocha* sp. nov.**
3	Wing with smoky pattern (Figs 37, 47)	**4**
–	Wing without smoky pattern (Figs 19, 28, 57)	**5**
4	Femora with two subapical rings (Fig. 37); prescutum with three longitudinal stripes (Fig. 36); wing dull brown without grayish spots at basal Rs, apical Sc, and R_2_ (Fig. 37); posterior margin of tergite 9 bearing a pair of finger-like protrusions (Fig. 39)	***L. subcosta* sp. nov.**
–	Femora with one subapical ring (Fig. 45); prescutum with one longitudinal stripe (Fig. 46); wings yellow with three grayish spots at basal Rs, apical Sc, and R_2_ (Fig. 47); posterior margin of tergite 9 without protrusions (Fig. 48)	***L. sylvicola* (Schummel, 1829)**
5	Body reddish brown (Fig. 55); wing brown with large spot at R_2_ (Fig. 57); prescutum with five longitudinal stripes (Fig. 56); gonostylus not swollen at base (Figs 58, 59)	***L. taurica* (Strobl, 1895)**
–	Body yellow (Figs 17, 26); wing pale brown with small spot at R_2_ (Figs 19, 28); prescutum with one longitudinal stripe (Figs 18, 27); gonostylus swollen at base (Figs 20, 29)	**6**
6	Wing with one obvious spot at R_2_ (Fig. 28); occiput yellow (Fig. 27); paramere with short apex (Fig. 32)	***L. stigma* (Meigen, 1818)**
–	Wing with three obvious dark spots (at basal Rs, apical Sc, and R_2_) (Fig. 19); occiput dark brown (Fig. 18); paramere with slender apex (Fig. 23)	***L. phragmitidis* (Schrank, 1781)**

### 1. Limonia macrostigma

Taxon classificationAnimaliaDipteraLimoniidae

(Schummel, 1829)

F03D58A4-1A44-5EDB-84E1-1616F83D1B77

Figures 1–3 , 4–7 , 8–9

Limnobia macrostigma Schummel 1829: 108.
Limonia alpicola Lackschewitz 1928: 231 (synonymy after Starý 2007).
Limonia (Limonia) venerabilis Alexander 1938: 134 (synonymy after Podenas and Podeniene 2017).
Limonia macrostigma Schummel: Savchenko 1985: 167; 1989: 333.
Limnobia macrostigma Schummel: Podenas and Podeniene 2017: 16 (redescription).

#### Diagnosis.

Flagellar verticils 2 times length of corresponding segment. Prescutum with three dark-brown longitudinal stripes. Wing pale brown with distinct, large, brown stigma; apical Sc_1_ slightly beyond base of Rs. Posterior margin of tergite 9 broadly emarginated. Paramere ending at 5/6 of aedeagus. Female, hypogynial valve 3.7 times longer than wide at base.

#### Redescription.

**Male** (*n* = 10): body length 7–9 mm, wing length 8–9.5 mm.

***Head*** mostly dark brown (Figs 6, 7). Vertex dark brown. Occiput dull yellow. Head with black setulae. Antenna dark brown and 14-segmented; pedicel oval; flagellomeres pale brown, nearly cylindrical; flagellar verticils black, 2 times longer than corresponding segment. Rostrum and nasus brown to dark brown. Nasus 3/5 as long as rostrum. Labella pale yellow except inner margin brown, with black setulae. Palpi black brown with black setulae, terminal two segments pale brown.

**Figures 1–3.**
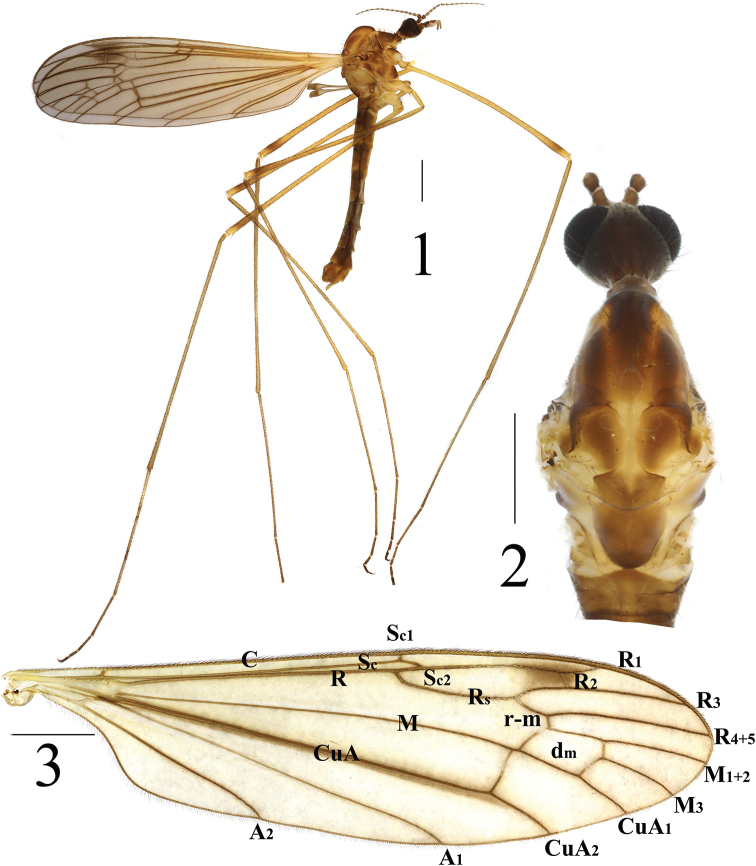
*Limonia macrostigma*, male **1** habitus, lateral view **2** head and thorax, dorsal view **3** right wing. Scale bars: 1.0 mm.

***Thorax*** mostly brown (Figs 1, 2). Cervical sclerite brown with black outer margin. Pronotum dull brown with black setulae. Prescutum with three dark-brown longitudinal stripes, median one with black setulae at outer margin. Scutum dark brown with black setulae at postero-lateral margin. Scutellum brown with tapered yellow median stripe. Mediotergite dark brown (Fig. 2). Mesopleura yellow to dark brown; subspiracular sclerite dull brown with pale-yellow spot at postero-lateral corner; episternum pale yellow to dark brown; katepisternum with black setulae and tapering, dark-brown marking at antero-lateral corner. Legs with coxae, trochanters yellow to pale brown except fore coxa yellow to dark brown; femora brown with one dark-brown, subapical ring; tibia brown; tarsi reddish brown to dark brown. Setulae on legs black. Wing hyaline, pale brown with large brown stigma at branching of R_1+2_; apical Sc_1_ slightly beyond base of Rs, Sc_2_ apically reaching 1/5 of Rs; basal deflection of CuA_1_ before branching point of M (Fig. 3). Halter: stem dull brown; knob bicolor, upper part pale brown to dark brown, lower part white (Fig. 1).

***Abdomen*** (Fig. 1). Mainly dull brown. Each abdominal segment with one pale yellow ring at posterior margin. Abdomen covered with golden setulae.

**Figures 4–7.**
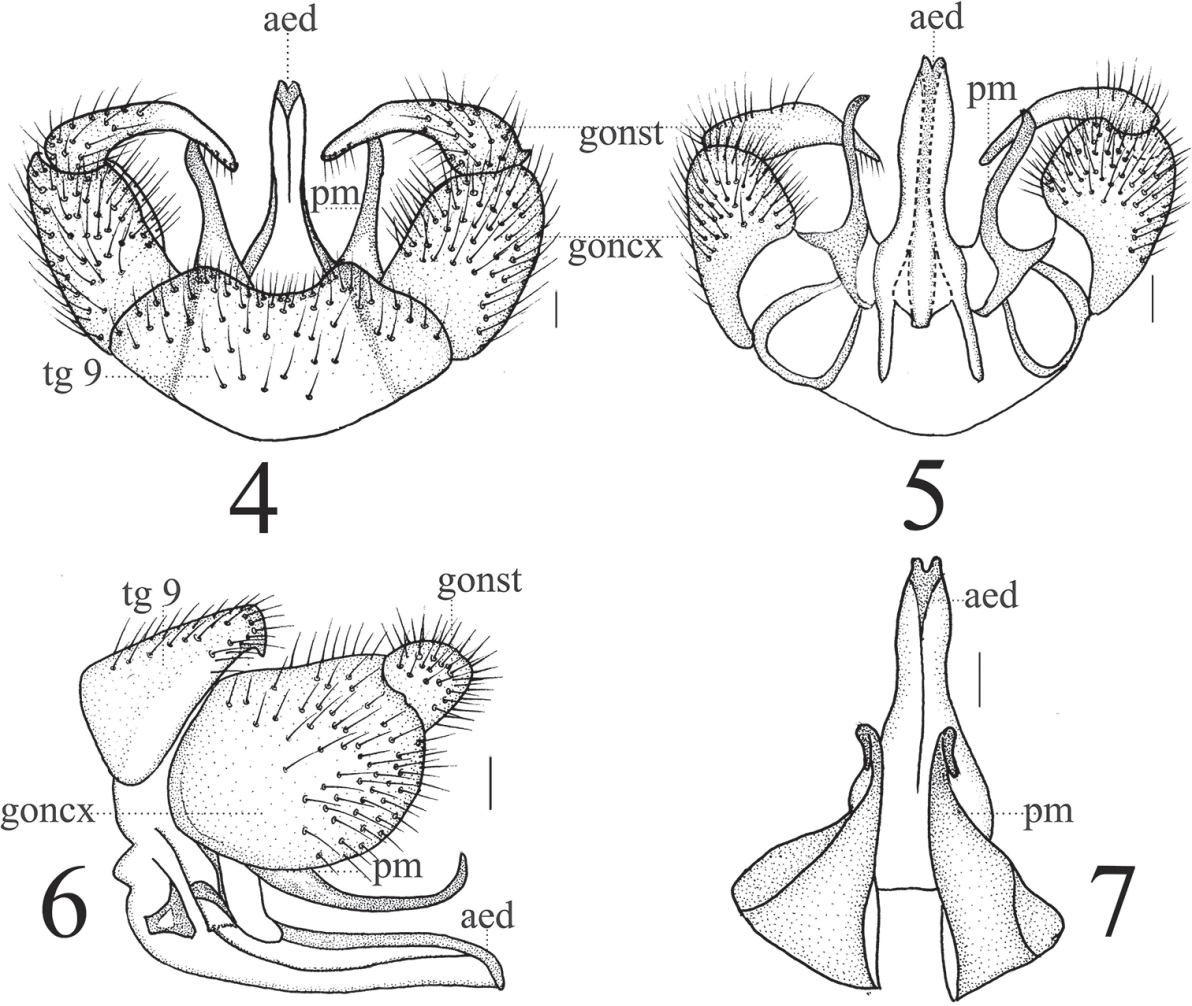
*Limonia macrostigma*, male **4** hypopygium, dorsal view **5** hypopygium, ventral view **6** hypopygium, lateral view **7** Aedeagus and paramere. Scale bars: 0.1 mm.

***Hypopygium*** dull brown (Figs 4–7). Posterior margin of tergite 9 broadly emarginated (Fig. 4). Gonocoxite weakly constricted at apex. Gonostylus with apex sharp, base swollen (Figs 4, 5). Paramere with fan-shaped base, very slender apex ending at 5/6 of aedeagus (Figs 4–7). Aedeagus forked; ventral mid-protrusion along middle line (Figs 4–7).

**Female** (*n* = 4): body length 7–8 mm, wing length 8–9 mm.

**Figures 8–9.**
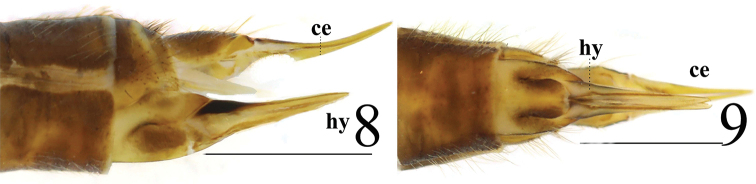
*Limonia macrostigma*, female **8** cercus and hypogynial valves, lateral view **9** cercus and hypogynial valves, ventral view. Scale bars: 1.0 mm.

**Female** resembling male in head, thorax and wing. Female terminalia dull brown. Cercus yellowish brown, slightly arched dorsally at apex, slender, 3 times longer than wide at base. Hypogynial valve 3.7 times longer than wide at base; lateral margin with triangular, black marking (Figs 8–9).

#### Material examined.

1 male, China: Xinjiang, Habahe, Celebaixiang, 48.08N, 86.331E, elev. 530 m, 2016.VII.9, Jinlong Ren (CAU). 7 males, 2 females, China: Xinjiang, Habahe, Baihabacun, 48.69N, 86.80E, elev. 1170 m, 2016.VII. 12, Jinlong Ren (light trap) (CAU). 12 males, 2 females, China: Xinjiang, Habahe, Baihabacun, 48.67N, 86.80E, elev. 1020 m, 2016.VII.12, Jinlong Ren (CAU). 8 males, 4 females, China: Xinjiang, Habahe, Baihabacun, 48.67N, 86.79E, elev. 1630 m, 2016.VII.12, Jinlong Ren (CAU). 2 males, 3 females, China: Xinjiang, Habahe, Baihabacun, 48.69N, 86.79E, elev. 1170 m, 2016.VII.12, Jinlong Ren (light trap) (CAU). 2 females (CAU), China: Xinjiang, Habahe, Baihabacun, 48.66N, 86.79E, elev. 1730 m, 2016.VII.13, Jinlong Ren (CAU). 1 male, China: Xinjiang, Burqin, Kanas, 48.68N, 86.99E, elev. 1470 m, 2016.VII.16, Jinlong Ren (CAU). 1 male, China: Xinjiang, Burqin, Kanas Lake, 48.74N, 87.01E, elev. 1390 m, 2016.VII.17, Jinlong Ren (CAU). 1 male, China: Xinjiang, Burqin, Kanas, 48.69N, 87.00E, elev. 1330 m, 2016.VII.18, Jinlong Ren (CAU). 2 males, China: Xinjiang, Burqin, Hemu, 48.58N, 87.45E, elev. 1160 m, 2016.VII.21, Jinlong Ren (CAU). 1 female, China: Xinjiang, Burqin, Hemu, 48.57N, 87.43E, elev. 1090 m, 2016.VII.22, Jinlong Ren (light trap) (CAU). 1 male, China: Xinjiang, Burqin, Hemu, 48.56N, 87.44E, elev. 1200 m, 2016.VII.23, Jinlong Ren (CAU).

#### Distribution.

Armenia, Austria, Azerbaijan, Belarus, Belgium, Bulgaria, China (Xinjiang: Burqin, Hababe), Croatia, Cyprus, Czech Rep., Denmark, Finland, France, Georgia, Germany, Great Britain, Greece, Hungary, Ireland, Italy, Kazakhstan, Kyrgyzstan. Latvia, Lithuania, Macedonia, Mongolia, Morocco, Netherlands, North Caucasus, North Korea, Norway, Pakistan, Poland, Romania, Russia, Serbia, Slovakia, Slovenia, Spain, Sweden, Switzerland, Tajikistan, Turkey, Turkey, Ukraine, Uzbekistan.

#### Remark.

This is the first report of this species from China. The position of Sc relative to Rs and female body color shows the geographic variation. First, specimens from Xinjiang (Burqin, Altay Mountain) have Sc_1_ slightly beyond basal Rs, Sc_2_ reaching 1/5 of Rs (Fig. 3), similar wing venation with Savchenko’s drawing (Savchenko 1985: 145, fig. 95.2), whereas specimens from Korea have Sc_1_ reaching 1/3 of Rs, Sc_2_ reaching 1/4 of Rs (Podenas and Podeniene 2017: 17, fig. 25). Moreover, female specimens from Xinjiang have the dark brown sternites, whereas Korean specimens have the yellow sternites (Podenas and Podeniene 2017:19).

### 2. Limonia medexocha sp. nov.

Taxon classificationAnimaliaDipteraLimoniidae

72F4E5DE-F8DF-5751-A0FF-C6D960138674

http://zoobank.org/5587C6AF-1509-4727-A3E1-452312C3254A

**Figures 10–12.**
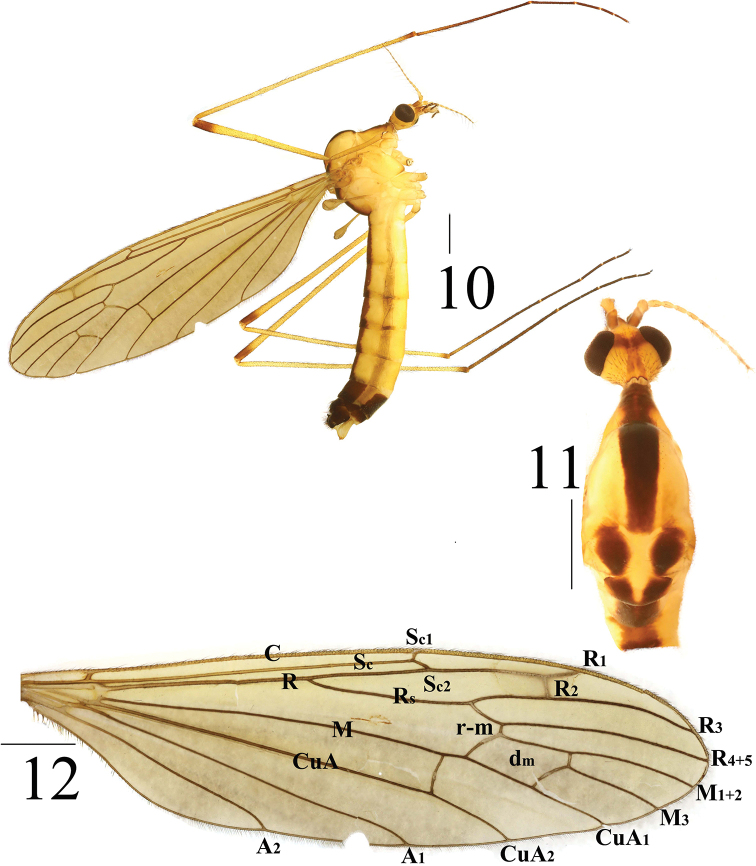
*Limonia medexocha* sp. nov., male **10** male habitus, lateral view **11** head and thorax, dorsal view **12** right wing. Scale bars: 1.0 mm.

**Figures 13–16.**
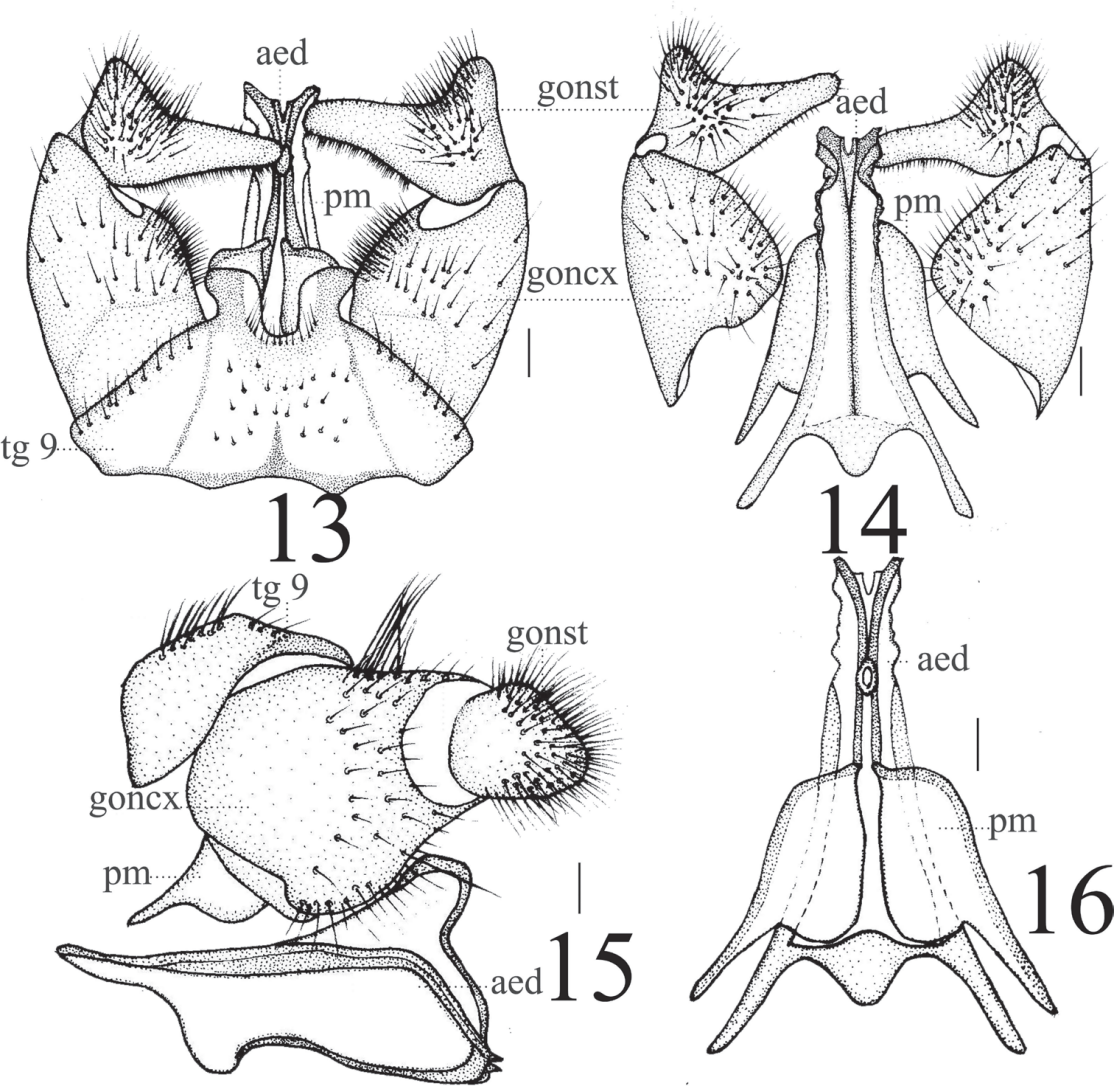
*Limonia medexocha* sp. nov., male **13** hypopygium, dorsal view **14** hypopygium, ventral view **15** hypopygium, lateral view **16** aedeagus and paramere. Scale bars: 0.1 mm.

Figures 10–12 , 13–16

#### Diagnosis.

Occipital marking Y-shaped. Flagellar verticils 3 times longer than corresponding segment. Postgena with short narrow stripe near inner margin of eyes. Prescutum with one broad, dark-brown mid-longitudinal stripe. Wing stigma pale brown with yellowish brown margin; Sc_1_ apically reaching 3/5 of Rs. Abdominal dorsum with one dark-brown mid-longitudinal stripe. Posterior margin of tergite 9 with two finger-like sclerotized protrusions. Gonostylus with blunt apex and swollen base. Paramere reniform, apex ended at 2/5 of aedeagus. Aedeagus with strong ventral mid-protrusion.

#### Description.

**Male** (*n* = 2): body length 9–10.5 mm, wing length 10–11 mm.

***Head*** mostly yellow (Figs 10, 11). Vertex dull yellow. Occiput dull yellow, covered with black setulae. Occipital marking dark brown, but pale brown at posterior part, Y-shaped, with anterior marking linked with inner margin of eyes. Head with black setulae. Antenna 14-segmented; scape dark brown, pedicel yellowish brown, flagellomeres yellowish brown; flagellar verticils black, three times longer than corresponding segment. Nasus 1/2 as long as rostrum. Rostrum and nasus brown to dark brown. Labella pale yellow, with black setulae and inner margin with brown. Postgena light yellow with short narrow stripe near inner margin of eyes. Palpi dark brown with black setulae.

***Thorax*** mostly yellow (Figs 10, 11). Cervical sclerite pale brown with black outer margin. Pronotum dull yellow with dull brown median stripe. Prescutum with one broad, dark-brown mid-longitudinal stripe and black setulae at lateral margin. Scutum dark brown with black setulae around outer margin. Scutellum dark brown with tapered yellow median stripe. Mediotergite dark brown (Fig. 11). Mesopleura entirely light yellow except katepisternum with dark brown margin at antero-lateral corner and subspiracular sclerite dull yellow; center of episternum with eight black setulae. Legs with coxae and trochanters yellow; femora yellowish brown with dark-brown terminal ring; tibiae yellowish brown, with dull brown subapical ring; tarsi reddish brown to dark brown. Setulae on legs black. Wing hyaline, pale brown; stigma pale brown with yellowish brown margin; Sc_1_ apically reaching 3/5 of Rs; while Sc_2_ apically reaching 3/4 of Rs; basal deflection of CuA_1_ far before branching point of M. (Fig. 12). Halter with dull-brown stem and pale-brown to dark-brown knob (Fig. 10).

***Abdomen*** mainly light yellow (Fig. 10). Dorsum with one dark-brown mid-longitudinal stripe. Abdominal segments 7–9 entirely dark brown; hypopygium mostly dark brown.

***Hypopygium*** (Figs 9–12). Posterior margin of tergite 9 with two finger-like, sclerotized protrusions that constrict at base and project outward at apex (Fig. 9). Gonocoxite wide at base and narrow at apex, longer than wide. Gonostylus with blunt apex and swollen basal protrusion that is strongly outward and hairy (Figs 9–11). Paramere reniform with small apical protrusion and rod-shaped; apex ended at 2/5 of aedeagus (Figs 13–16). Aedeagus complex; mid-ventral margin with strong protrusion which is long and rectangular in lateral view (Fig. 15); outer margin serrated; apex with small notch; spherical protrusion located mid-ventrally at base (Figs 13–16).

**Figures 17–19.**
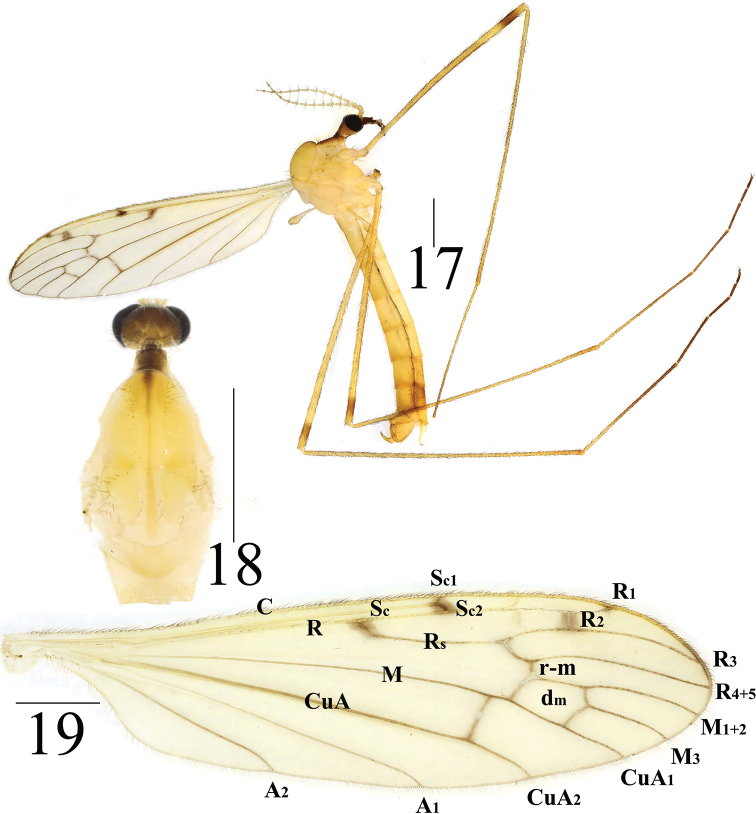
*Limonia phragmitidis*, male **17** male habitus, lateral view **18** head and thorax, dorsal view **19** right wing. Scale bars: 1.0 mm.

**Female** (unknown)

#### Type material.

***Holotype*** male, China: Ningxia, Guyuan, Liupan Mountain, 35.38N, 106.31E, elev. 2210 m, 2017.VII.20, Jiale Zhou (CAU). ***Paratypes***: 1 male, China: Sichuan, Pingwu, Wanglang, elev. 2910 m, 2017.VII.29, Yuqiang Xi (CAU).

#### Distribution.

China (Ningxia: Guyuan; Sichuan: Pingwu).

#### Etymology.

The specific name, from Latin, *medius* (adj., meaning “middle”), and Greek *exocha* (adj., meaning “protruded”), refers to the median protrusion at the dorsal margin of the aedeagus.

#### Remarks.

This new species is very unique and differs from other known species of *Limonia*. This new species is somewhat similar to *L. macrostigma* (Schummel, 1829) in wing stigma. It can be separated from the latter by the following features: body yellow; occiput yellow with Y-shaped marking; gonostylus with obtuse apex; paramere with obtuse apex; aedeagus with strong mid-ventral protrusion. In *L. macrostigma*, the body is brown; the occiput is black brown; the prescutum has two lateral stripes; the gonostylus has a slender apex; the paramere has a slender and pointed apex; and the aedeagus has no mid-dorsal protrusion (Podenas and Podeniene 2017).

### 3. Limonia phragmitidis

Taxon classificationAnimaliaDipteraLimoniidae

(Schrank, 1781)

C43CB0EE-0929-51D4-9E4A-9106D769E4BE

Figures 17–19 , 20–23 , 24–25

Tipula phragmitidis Schrank 1781: 605.
Tipula tripunctata Fabricius 1781: 405 (subsequent designation by Westwood 1840).
Tipula phragmitidis Schrank: Kolcsár et al. 2017: 50 (redescription).

#### Diagnosis.

Flagellar verticils shorter than corresponding segment. Pronotum dark brown. Prescutum with one narrow, deep-brown longitudinal stripe. Wing pale brown with three small, brown markings at base of Rs, apical Sc, and R_2_; Sc_1_ apically reaching 1/2 of Rs. Gonostylus with sharp apex and swollen base. Paramere ended at 3/5 of aedeagus. Aedeagus with unique H-shaped pattern at the mid-ventral margin. Female, hypogynial valve 2.1 times longer than wide at base.

#### Redescription.

Male (*n* = 7): body length 7–8.5 mm, wing length 8–9 mm.

***Head*** dull brown (Figs 17, 18). Vertex dull brown. Occiput dull yellow, covered with black setulae. Antenna yellow and 14-segmented; scape yellow; pedicel oval; flagellomeres nearly cylindrical; flagellar verticils black, shorter than corresponding segment. Rostrum brown or dark brown. Labella pale yellow, except inner margin brown covered with black setulae. Palpi brown with black setulae.

***Thorax*** mostly yellow (Figs 17, 18). Cervical sclerite pale brown, with black outer margin. Pronotum dark brown with black setulae. Prescutum with rather narrow, brown longitudinal stripe (anterior part wider than posterior part). Scutum pale yellow, with black setulae around outer margin. Scutellum pale yellow. Mediotergite pale yellow to yellow (Fig. 18). Mesopleura pale yellow to yellow; subspiracular sclerite pale yellow; anepimeron and katepisternum with black setulae. Legs with coxae and trochanters pale yellow; femora yellow with one dark-brown subapical ring; tibiae brown; tarsi brown to dark brown. Setulae on legs black. Wing hyaline, pale brown, with three small brown markings at base of R_s_, apical Sc, and R_2_; Sc_1_ apically reaching 1/2 of Rs; basal deflection of CuA_1_ before branching point of M (Fig. 19). Halter: stem white; knob bicolor with upper part pale yellow to yellow while lower part white (Fig. 17).

***Abdomen*** mainly yellow (Fig. 17). Sternite 8 reddish brown. Each abdominal segment with one pale-brown ring at posterior margin. Abdomen covered with golden setulae.

**Figures 20–23.**
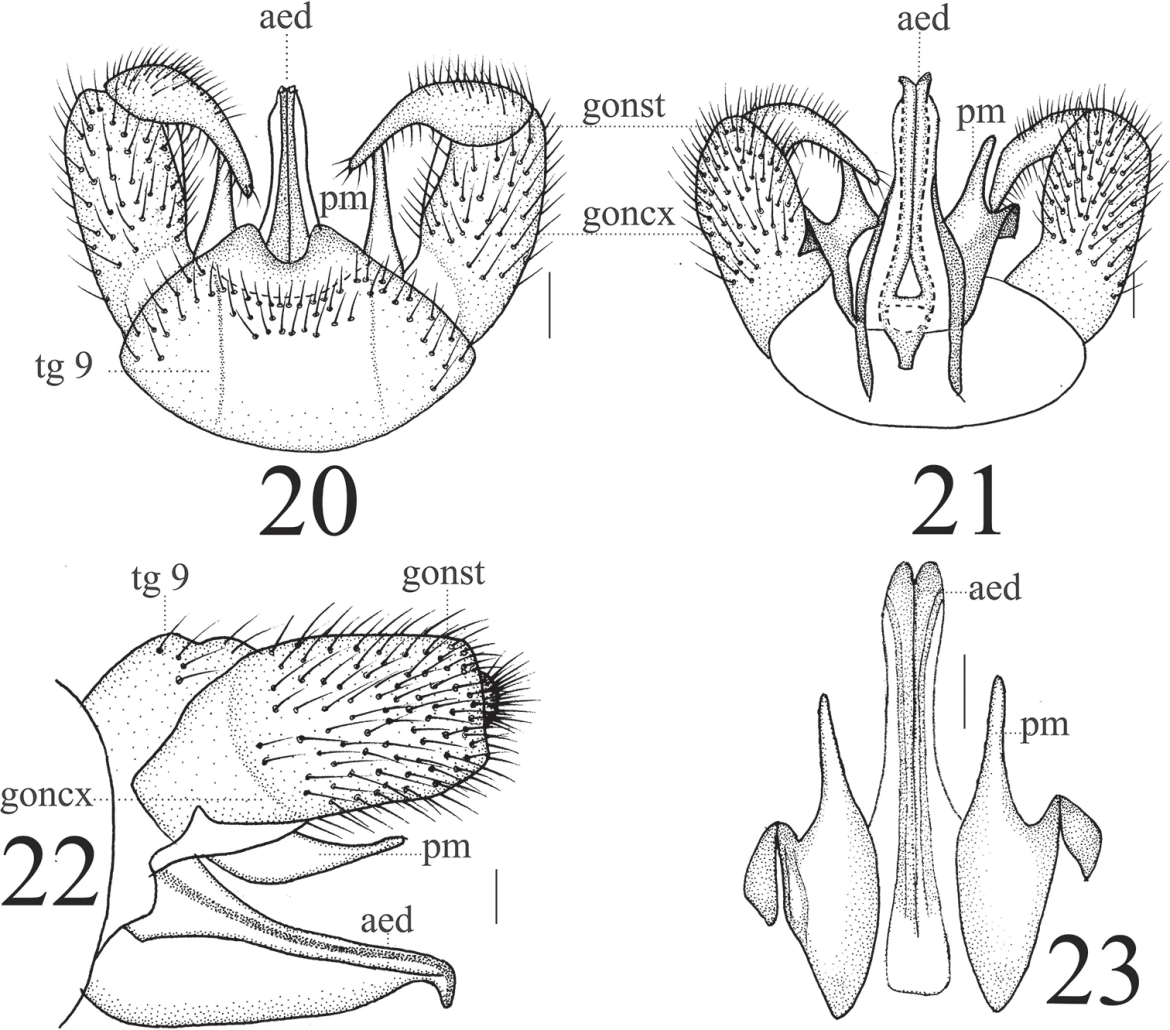
*Limonia phragmitidis*, male **20** hypopygium, dorsal view **21** hypopygium, ventral view **22** hypopygium, lateral view **23** aedeagus and paramere. Scale bars: 0.1 mm.

***Hypopygium*** pale yellow (Figs 20–23). Posterior margin of tergite 9 narrowly emarginated (Fig. 20). Gonocoxite long cylindrical. Gonostylus with sharp apex and swollen base that covered with black setulae (Figs 20, 21). Paramere with fan-shaped base and sharp apex that ends at 3/5 of aedeagus (Figs 20–23). Aedeagus forked; ventral margin with mid-protrusion that triangular in lateral view and unique H-shaped pattern in middle (Figs 20–23).

**Female** (*n* = 6): body length 8–10 mm, wing length 8–10 mm.

**Figures 24–25.**
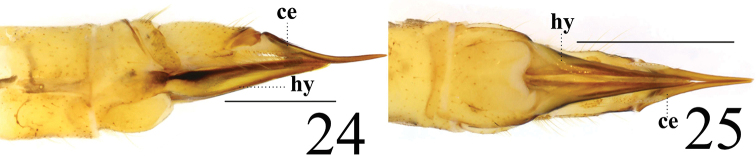
*Limonia phragmitidis*, female **24** cercus and hypogynial valves, lateral view **25** cercus and hypogynial valves, ventral view. Scale bars: 1.0 mm.

**Female** resembling male in head, thorax, and wing. Female terminalia pale yellow. Cercus yellowish brown, slightly arched dorsally at apex, slender, and 2.3 times longer than wide at base. Hypogynial valve 2.1 times longer than wide at base; lateral margin with black marking (Figs 24, 25).

#### Material examined.

1 male, China: Xinjiang, Habahe, Baihabacun, 48.69N, 86.79E, elev. 1170 m, 2016.VII.12, Jinlong Ren (light trap) (CAU). 24 males, 20 females, China: Xinjiang, Burqin, Hemu, 48.43N, 87.57E, elev. 1040 m, 2016.VII.21, Jinlong Ren (CAU). 60 males, 40 females, China: Xinjiang, Burqin, Hemu, 48.58N, 87.45E, elev. 1160 m, 2016.VII.21, Jinlong Ren (CAU). 47 males, 37 females, China: Xinjiang, Burqin, Hemu, 48.56N, 87.44E, elev. 1150 m, 2016.VII.22, Jinlong Ren (CAU). 35 males, 30 females, China: Xinjiang, Burqin, Hemu, 48.56N, 87.44E, elev. 1210 m, 2016.VII.23, Jinlong Ren (CAU). 1 female, China, Xinjiang, Gongliu, Kuerdening, 43.28N, 82.95E, elev. 1270 m, 2017.VII.26, Bing Zhang (CAU). 1 female, China, Xinjiang, Gongliu, Kuerdening, 43.25N, 82.83E, elev. 1140 m, 2017.VII.26, Bing Zhang (light trap) (CAU).

#### Distribution.

Albania, Andorra, Austria, Azerbaijan, Belarus, Belgium, Bosnia-Herzegovina, Bulgaria, China (Xinjiang: Burqin, Habahe, Gongliu), Croatia, Czech Rep., Denmark, Finland, France, Georgia, Germany, Great Britain, Greece, Hungary, Ireland, Israel, Italy, Jordan, Kazakhstan, Kyrgyzstan, Latvia, Lithuania, Luxembourg, Macedonia, Montenegro, Morocco, Netherlands, North Caucasus, Norway, Poland, Romania, Russia, Serbia, Slovakia, Slovenia, Spain, Sweden, Switzerland, Turkey, Turkey, Ukraine.

#### Remark.

This is the first report of this species from China.

### 4. Limonia stigma

Taxon classificationAnimaliaDipteraLimoniidae

(Meigen, 1818)

3A177995-18CD-55C0-AAA4-5BFAFED2B8AA

Figures 26–28 , 29–32 , 33–34

Limnobia stigma Meigen 1818: 138.
Limnobia terrestris Linnaeus 1758: 586 (synonymy).
Limnobia sexnotata Schumme 1829: 111 (synonymy).
Limnobia punctigera Walker 1856: 298 (synonymy).

#### Diagnosis.

Flagellar verticils black, 1.6 times longer than corresponding segment. Prescutum with one triangular, brown longitudinal stripe. Wing pale brown, with one small grayish black marking at R_2_; Sc_1_ apically reaching 1/2 of Rs. Gonocoxite long cylindrical. Gonostylus with sharp apex and swollen, hairy base. Paramere with fan-shaped base and blunt apex that ends at 1/2 of aedeagus. Aedeagus with Y-shaped pattern at mid-ventral margin. Female, hypogynial valve 2.3 times longer than wide at base.

#### Redescription.

Male (*n* = 19): body length 7–9 mm, wing length 8–9 mm.

**Figures 26–28.**
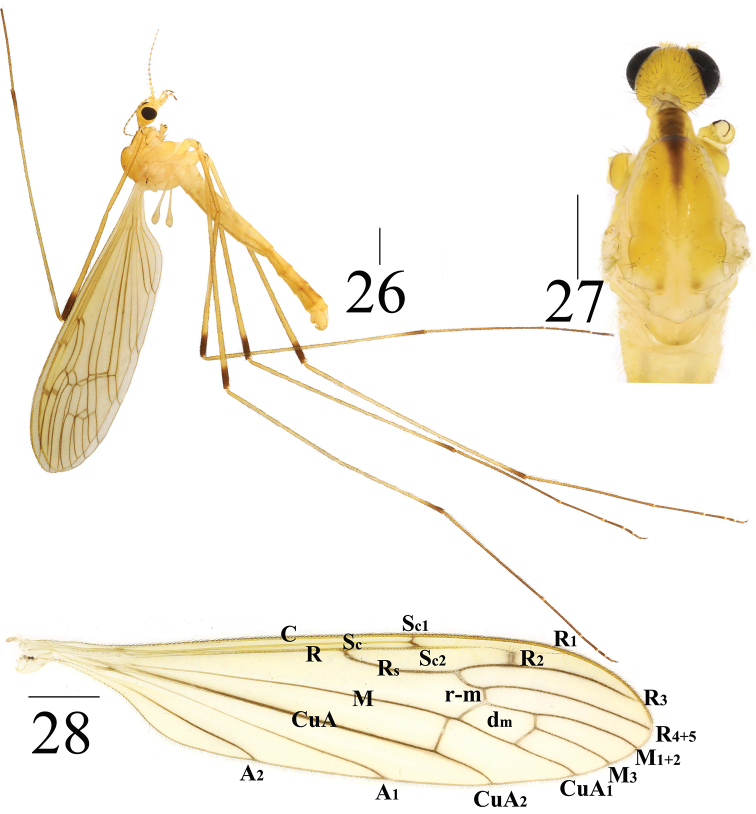
*Limonia stigma*, male **26** male habitus, lateral view **27** head and thorax, dorsal view **28** right wing. Scale bars: 1.0 mm.

***Head*** yellow (Figs 26–28). Vertex yellow. Occiput dull yellow, covered with black setulae. Antenna yellow and 14-segmented; scape yellow; pedicel oval; flagellomeres cylindrical and bicolor, brown at base and pale yellow at apex; flagellar verticils black, 1.6 times longer than corresponding segment. Rostrum and nasus pale yellow, nasus 1/4 as long as rostrum. Labella pale yellow, with black setulae, except inner margin brown. Palpi yellow except terminal segment brown.

***Thorax*** mostly yellow (Figs 17, 18). Cervical sclerite yellow with black margin at antero-lateral margin. Pronotum dark brown, with black setulae. Prescutum with one brown, triangular longitudinal stripe (anterior part wider than posterior part); two setulae and longitudinal rows at middle. Scutum pale yellow, with black, oblique setulae at middle. Scutellum white. Mediotergite pale yellow, variegated with white (Fig. 27). Mesopleura pale yellow to yellow; subspiracular sclerite yellow; katepisternum with black setulae. Legs with coxae and trochanters yellow; femora dull yellow with one dark-brown subapical ring; tibiae brown with dark-brown subapical ring; tarsi brown to dark brown. Setulae on legs black. Wing hyaline, pale brown with one small grayish black marking at R_2_; Sc_1_ apically reaching 1/2 of Rs; basal deflection of CuA_1_ before branching point of M (Fig. 28). Halter dull yellow; knob bicolor, with upper part dull yellow and lower part white (Fig. 26).

***Abdomen*** mainly yellow (Fig. 26). Sternite 7 reddish brown. Each abdominal segment with one pale-brown ring at posterior margin. Abdomen covered with golden setulae.

**Figures 29–32.**
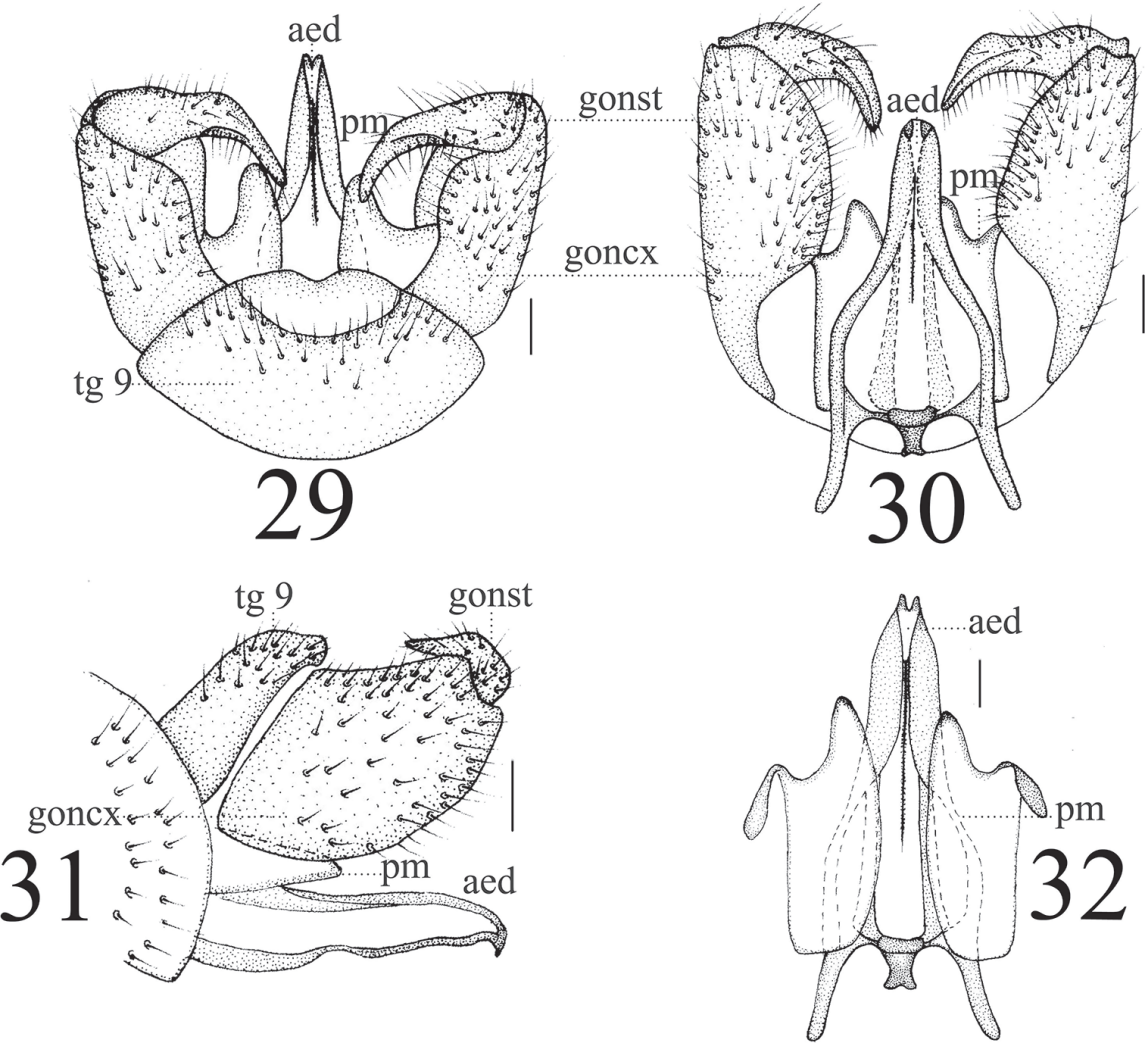
*Limonia stigma*, male **29** hypopygium, dorsal view **30** hypopygium, ventral view **31** hypopygium, lateral view **32** aedeagus and paramere. Scale bars: 0.1 mm.

***Hypopygium*** yellow (Fig. 26). Posterior margin of tergite 9 emarginated (Fig. 29). Gonocoxite long cylindrical. Gonostylus with sharp apex and swollen and hairy base (Figs 29, 30). Paramere with fan-shaped base and blunt apex that ended at 1/2 of aedeagus (Figs 29–32). Aedeagus forked; ventral margin with mid-protrusion triangular in lateral view; unique Y-shaped pattern at middle that anterior margin with short mid-protrusion (Figs 20–23).

**Female** (*n* = 11): body length 7.5–9 mm, wing length 7.5–10 mm.

**Figures 33–34.**
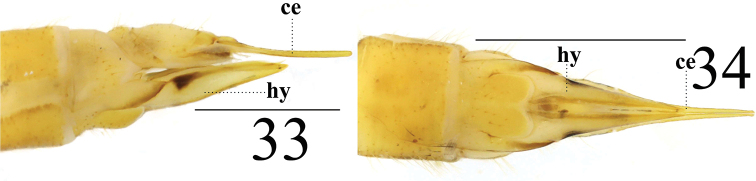
*Limonia stigma*, female **33** cercus and hypogynial valves, lateral view **34** cercus and hypogynial valves, ventral view. Scale bars: 1.0 mm.

**Female** resembling male in head, thorax, and wing. Female terminalia pale yellow. Cercus yellowish brown, slightly arched dorsally at apex, slender, and 3 times longer than wide at base. Hypogynial valve 2.3 times longer than wide at base; lateral margin with triangular black marking (Figs 33, 34).

#### Material examined.

6 males, China: Xinjiang, Burqin, Hemu, 48.43N, 87.57E, elev. 1040 m, 2016.VII.21, Jinlong Ren (CAU). 13 males, 2 females, China: Xinjiang, Burqin, Hemu, 48.58N, 87.45E, elev. 1160 m, 2016.VII.21, Jinlong Ren (CAU). 32 males, 16 females, China: Xinjiang, Burqin, Hemu, 48.56N, 87.44E, elev. 1150 m, 2016.VII.22, Jinlong Ren (CAU). 10 males, 3 females, China: Xinjiang, Burqin, Hemu, 48.56N, 87.44E, elev. 1200 m, 2016.VII.23, Jinlong Ren (CAU).

#### Distribution.

Armenia, Austria, Belarus, Belgium, Bulgaria, China (Xinjiang: Burqin), Czech Rep., Denmark, Estonia, Finland, France, Germany, Great Britain, Hungary, Italy, Lithuania, Netherlands, Poland, Romania, Russia, Slovakia, Slovenia, Sweden, Switzerland, Ukraine.

#### Remark.

This is the first report of this species from China.

### 5. Limonia subcosta sp. nov.

Taxon classificationAnimaliaDipteraLimoniidae

482D6AD4-8234-5659-99F3-29EF7116C57E

http://zoobank.org/142913C6-D932-4AE5-8169-D44C8D3EE2AF

Figures 35–37 , 39–42 , 43–44

#### Diagnosis.

Flagellar verticils 1.5 times longer than corresponding segment. Prescutum with three reddish-brown longitudinal stripes. Scutum dark brown, with triangular yellow marking at postero-lateral margin. Wing dull brown, variegated with zigzag whitish bands at origin of Rs before cord; Sc_2_ apically reaching 1/2 of Rs. Posterior margin of tergite 9 emarginated with two finger-like, sclerotized protrusions. Paramere with blunt apex that ends at 7/10 of aedeagus. Female, hypogynial valve 1.8 times longer than wide at base.

#### Description.

**Male** (*n* = 4): body length 6.5–7 mm, wing length 7–7.5 mm.

**Figures 35–38.**
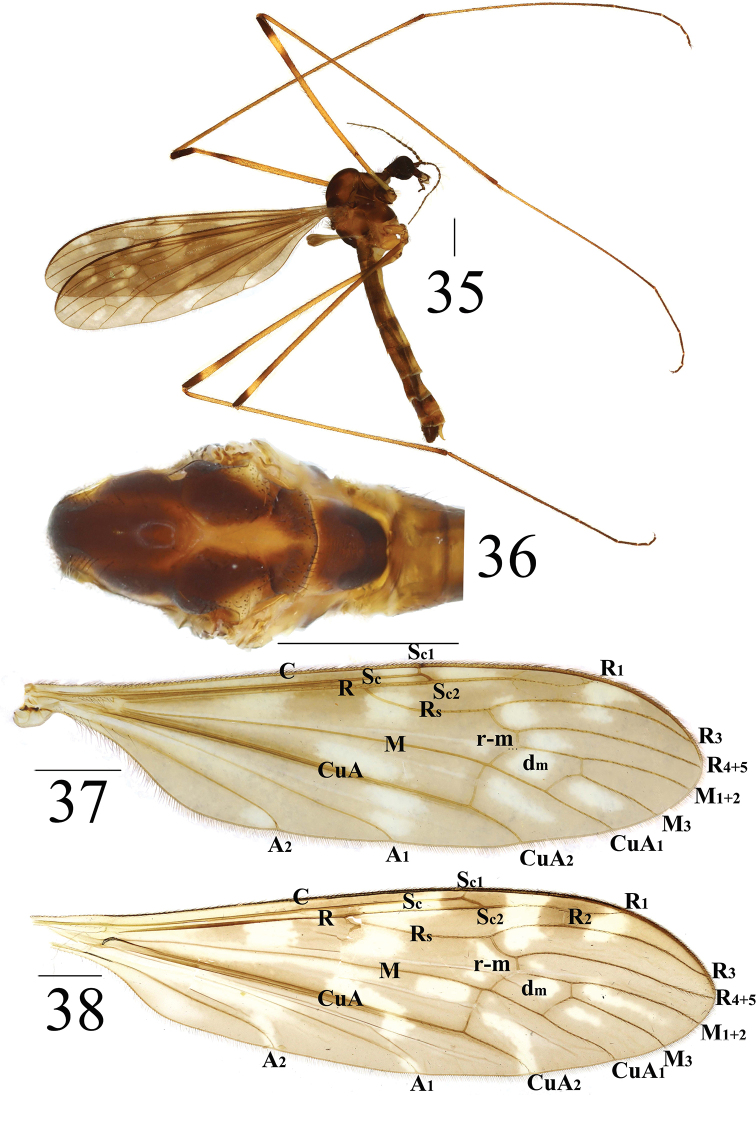
*Limonia subcosta* sp. nov., male **35** male habitus, lateral view **36** head and thorax, dorsal view **37** right wing **38** wing of *Limonia pernigrina* Alexander, 1938, holotype. Scale bars: 1.0 mm.

***Head*** mostly black-brown (Fig. 35). Vertex dark brown. Occiput dark brown, covered with sparse setulae. Antenna 14-segmented; scape, pedicel, and flagellomeres black-brown, except for basally dull-yellow first flagellomere; flagellar verticils black, 1.5 times longer than corresponding segment. Nasus 2/5 as long as rostrum. Rostrum and nasus brown to dark brown, with black setulae. Labella pale brown, with black setulae. Palpi brownish gray, with black setulae.

***Thorax*** (Figs 35, 36). Mostly reddish brown to black brown. Cervical sclerite dark brown, with black outer margin. Pronotum dark brown, with sparse black setulae. Prescutum with three reddish brown longitudinal stripes. Scutum dark brown, with triangular yellow marking at postero-lateral margin. Scutellum brown with black setulae at posterior margin. Mediotergite reddish brown, with U-shaped, black-brown marking at posterior margin (Fig. 28). Mesopleura entirely black-brown; episternum with sparse, yellow setulae. Legs: coxae and trochanters brown, with black setulae; femora, tibiae, and tarsi dull brown; femora with two subapical rings (outer one black-brown, inner one dull brown). Wing dull brown, variegated with zigzag whitish bands at origin of Rs before cord (basal section of R_4+5_, r-m, and m-cu); Sc_2_ apically reaching 1/2 of Rs; basal deflection of CuA_1_ slightly beyond branching point of M; R_2_ absent (Fig. 37). Halter with stem brown; knob dull brown (Fig. 35).

***Abdomen*** (Fig. 35). Mainly reddish brown. Posterior margin of abdominal segments 1–4 with dark-brown ring. Venter dull yellow. Posterior margin of abdominal segments 5–8 with pale yellow ring. Hypopygium reddish brown. Abdominal setulae black.

**Figures 39–42.**
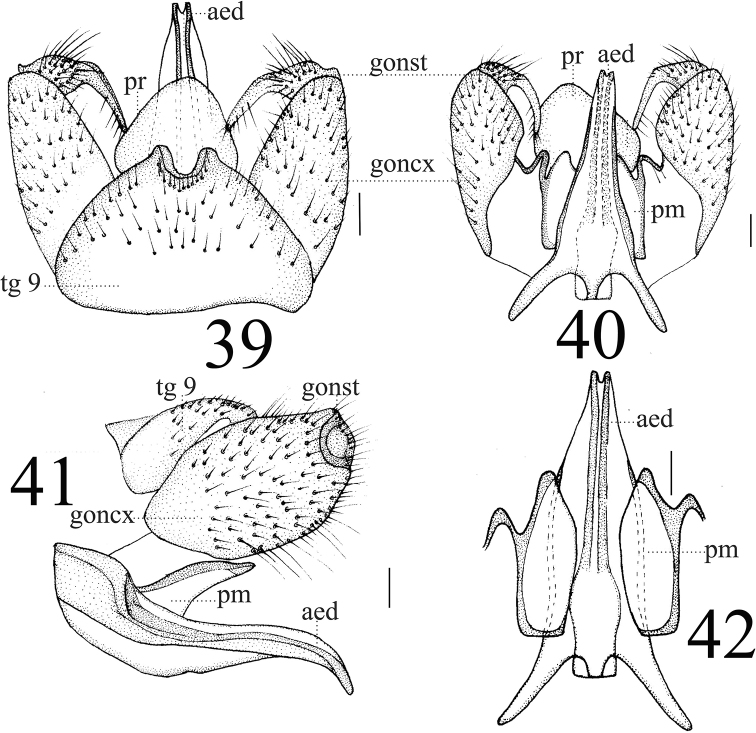
*Limonia subcosta* sp. nov., male **39** hypopygium, dorsal view **40** hypopygium, ventral view **41** hypopygium, lateral view **42** aedeagus and paramere. Scale bars: 0.1 mm.

***Hypopygium*** (Figs 39–42). Posterior margin of tergite 9 emarginated with two finger-like, sclerotized protrusions (Fig. 39). Gonocoxite wider than long (Figs 39–41). Gonostylus black-brown, apically slender, and basally with slightly swollen covered with longer setulae (Figs 31, 32). Proctiger globular and membranous. Paramere with short obtuse apex (Fig. 34). Paramere with fan-shaped base and blunt apex that ended at 7/10 of aedeagus (Figs 41, 42). Aedeagus forked; ventral margin with mid-protrusion that anterior margin with cube-shaped (Figs 40, 42).

**Female** (*n* = 5): body length 7–8 mm, wing length 7–8.5 mm.

**Figures 43–44.**
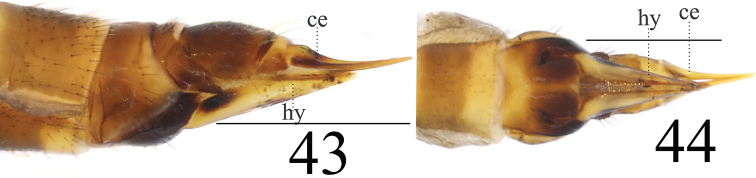
*Limonia subcosta* sp.nov., female **43** cercus and hypogynial valves, lateral view **44** cercus and hypogynial valves, ventral view. Scale bars: 1.0 mm.

**Female** resembling male in head, thorax, and wing. Female terminalia (Fig. 46) reddish brown. Cercus brown, with slightly arched dorsally at apex, slender, and 2 times longer than wide at base. Hypogynial valve 1.8 times longer than wide at base; lateral margin with oval, black marking (Figs 43, 44).

#### Type material.

***Holotype*** male, China: Shaanxi, Foping, Panda valley, 33.66N, 107.98E, elev. 1470 m, 2016.VII.10–2016.VII.21, Ruie Nie (Malaise trap) (CAU). ***Paratypes***: 2 males, 2 females, China: Shaanxi, Foping, Panda valley, 33.67N, 107.98E, elev. 1460 m, 2016.VII.17–2016.VII.19, Ruie Nie (Malaise trap) (CAU). 1 male, China: Shaanxi, Foping, Panda valley, 33.67N, 107.979E, elev. 1470 m, 2016.VII.17, Ruie Nie (Malaise trap) (CAU). 1 male, 1 female, China: Shaanxi, Foping, Panda valley, 33.67N, 107.98E, elev. 1470 m, 2016.VII.17–2016. VII.21, Ruie Nie (Malaise trap) (CAU). 2 females, China: Shaanxi, Yangxian, Maopingzhen, elev. 910 m, 2017. VIII.8, Xulong Chen (Malaise trap) (CAU).

#### Distribution.

China (Shaanxi: Foping, Yangxian).

#### Etymology.

The specific name, from Latin, *sub* and *costa* (meaning “below the costa”, refers to relative position of Sc to Rs.

#### Remarks.

This new species is similar to *L. pernigrina* Alexander, 1938 in the wing marking and shape of the gonostylus. It can be separated from the latter by the following features: Sc_2_ ended at 1/2 of Rs; branch pointing of Rs with small, whitish spot; posterior margin of tergite 9 with a narrow, median recession and long, finger-like protrusions (Figs 29, 31). In *L. pernigrina*, the Sc_2_ ends almost at branch of Rs; the branch pointing of Rs has a large whitish band that is linked anteriorly with the costal margin of the wing (Fig. 30); and the posterior margin of tergite 9 has a broad, median recession and short, finger-like protrusions (Alexander 1938b).

### 6. Limonia sylvicola

Taxon classificationAnimaliaDipteraLimoniidae

(Schummel, 1829)

BC62FF5A-CF77-5834-B655-E3A056384B12

Figures 45–47 , 48–52 , 53

Limnobia sylvicola Schummel 1829: 605.
Limnobia affinis Zetterstedt 1838: 605 (synonymy).
Limnobia tripunctata Zetterstedt 1838: 605 (synonymy).

#### Diagnosis.

Flagellar verticils 2 times longer than corresponding segment, but shorter in some specimens. Prescutum with one very broad brown mid-longitudinal stripe. Wings yellow variegated with grayish smoky markings, and three grayish spots at basal Rs, apical Sc and R_2_; Sc_1_ apically reaching 1/2–3/4 of Rs. Gonostylus long and slender. Posterior margin of tergite 9 emarginated. Gonostylus with sharp apex and swollen base. Apex of paramere ended at 3/4 of aedeagus. Ventral margin of aedeagus with H-shaped. Female, hypogynial valve 2.3 times longer than wide at base.

#### Redescription.

Male (*n* = 13): body length 6–8 mm, wing length 7–8 mm.

**Figures 45–47.**
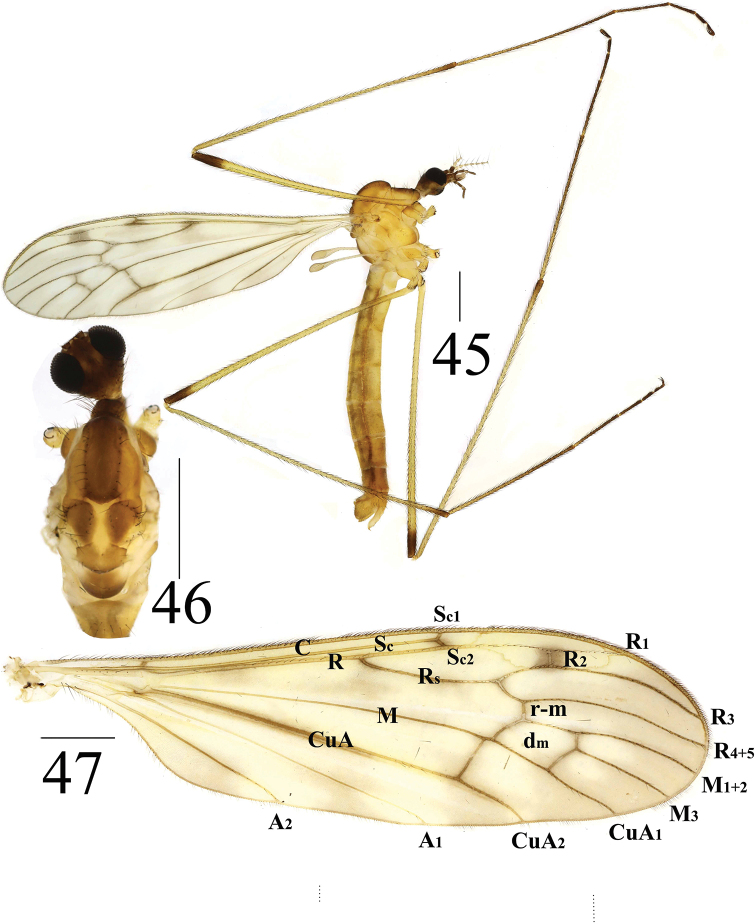
*Limonia sylvicola*, male **45** male habitus, lateral view **46** head and thorax, dorsal view **47** right wing. Scale bars: 1.0 mm.

***Head*** dull brown (Figs 45, 46). Vertex dull brown, with two yellow markings at outer margin near eyes (Fig. 46). Occiput dull yellow, covered with black setulae. Antenna yellow and 14-segmented; scape and pedicel dull brown; flagellomeres nearly cylindrical; flagellar verticils grayish yellow, 2 times longer than corresponding segment but short in some specimens. Nasus 1/2 as long as rostrum. Rostrum brown or dull brown. Labella pale yellow, except inner margin brown. Palpi brown, with black setulae.

***Thorax*** mostly brownish yellow (Figs 45, 46). Cervical sclerite pale brown, with black outer margin. Pronotum dark brown, with black setulae. Prescutum with broad, brown longitudinal stripe, and with black setulae at lateral margin. Scutum brown, with black setulae at outer margin. Scutellum pale yellow, but mid-anterior margin with round, yellow marking. Mediotergite dull brown (Fig. 46). Mesopleura dull yellow; subspiracular sclerite brownish yellow; anepimeron and katepisternum with black setulae. Legs with coxae and trochanters yellow, except frontal coxae brown; femora yellow, with one dark-brown subapical ring; tibiae yellow, with reddish-brown subapical ring; tarsi yellow to dark brown onwards. Setulae on legs black. Wings yellow, variegated with grayish smoky markings, and three grayish spots at basal Rs, apical Sc, and R_2_; Sc_1_ apically reaching 1/2–3/4 of Rs; basal deflection of CuA_1_ beyond branching point of M; some specimens R_2_ absent in some specimens (Fig. 47). Halter: stem white; knob dull gray.

***Abdomen*** mainly brown (Fig. 45). Sternite 8 reddish brown. Each abdominal segment with one pale-brown ring at posterior margin. Abdomen covered with golden setulae. Abdominal segments 7–8 reddish brown.

**Figures 48–52.**
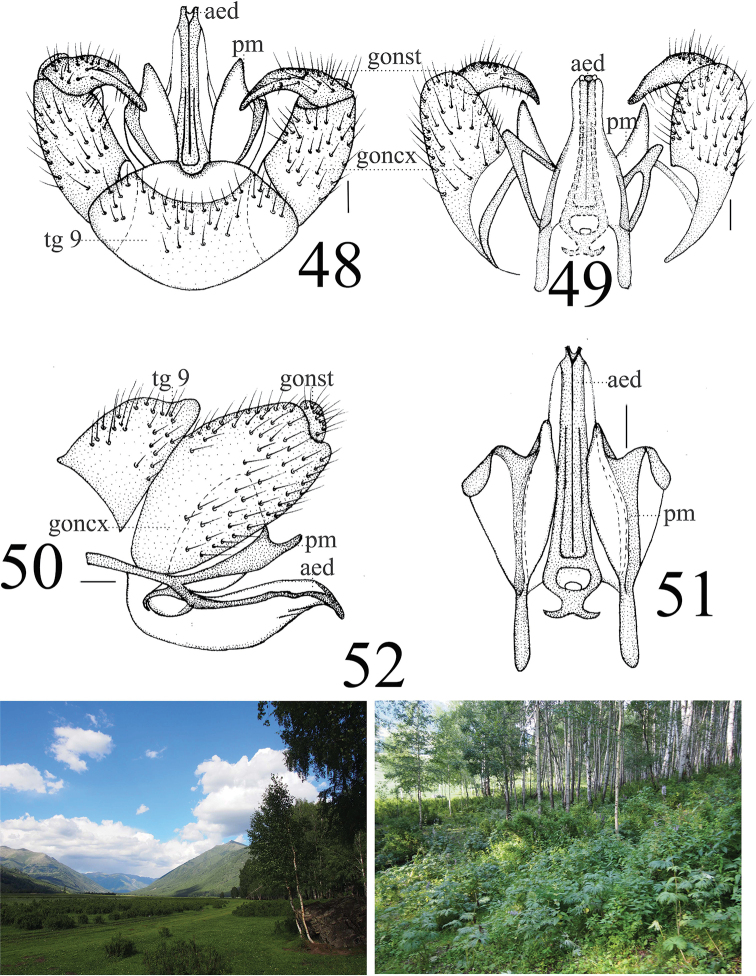
*Limonia sylvicola*, male **48** hypopygium, dorsal view **49** hypopygium, ventral view **50** hypopygium, lateral view **51** aedeagus and paramere **52** habitat, Hemu, Burqin, Xinjiang, China on 22 June 2017. Scale bars: = 0.1 mm (48–51).

***Hypopygium*** reddish brown (Figs 49–51). Posterior margin of tergite 9 emarginated (Fig. 48). Gonocoxite long cylindrical. Gonostylus with sharp apex and swollen base covered with black setulae (Figs 48, 49). Paramere with fan-shaped base and sharp apex, ending at 3/4 of aedeagus (Figs 49–51). Aedeagus forked; ventral margin with mid-protrusion triangular in lateral view; ventral margin with uniquely H-shaped pattern in middle and anterior margin fish-tail-like in shape (Figs 49, 51).

**Female** (*n* = 4): body length 9 mm, wing length 8–10 mm.

**Figures 53–54.**
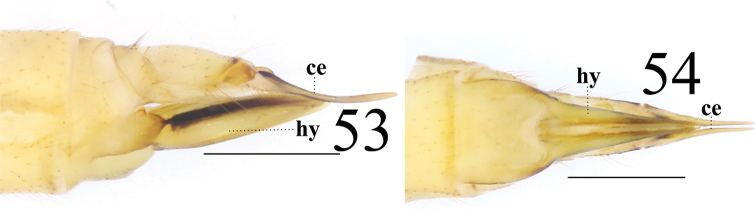
*Limonia sylvicola*, female **53** cercus and hypogynial valves, lateral view **54** cercus and hypogynial valves, ventral view. Scale bars: 1.0 mm.

**Female** resembling male in head, thorax, and wing. Female terminalia pale yellow. Cercus yellow with slightly arched dorsally at apex, slender and 2.4 times longer than wide at base. Hypogynial valve 2.3 times longer than wide at base; lateral margin with broad, black marking (Figs 53, 54).

#### Biology.

This species is often collected with *L. phragmitidis* (Schrank, 1781) and *L. stigma* (Meigen, 1818), and it is a dominant species in Hemu (China: Xinjiang, Burqin). These species often aggregate in shaded understory plants in white birch forests during the day (Fig. 46).

#### Material examined.

1 male, China: Xinjiang, Habahe, Baihabacun, 48.68N, 86.79E, elev. 1350 m, 2016.VII.11, Jinlong Ren (CAU). 1 male, China: Xinjiang, Habahe, Baihabacun, 48.67N, 86.80E, elev. 1020 m, 2016.VII.12, Jinlong Ren (CAU). 1 male, China: Xinjiang, Habahe, Baihabacun, 48.67N, 86.79E, elev. 1630 m, 2016.VII.12, Jinlong Ren (CAU). 1 male China: Xinjiang, Habahe, Baihabacun, 48.66N, 86.79E, elev. 1730 m, 2016.VII.13, Jinlong Ren (CAU). 2 males, China: Xinjiang, Burqin, Kanas, 48.68N, 86.99E, elev. 1470 m, 2016.VII.16, Jinlong Ren (CAU). 7 males, 2 females, China: Xinjiang, Burqin, Kanas Lake, 48.74N, 87.01E, elev. 1390 m, 2016.VII.17, Jinlong Ren (CAU). 2 males, China: Xinjiang, Burqin, Kanas, 48.66N, 87.00E, elev. 1310 m, 2016.VII.19, Jinlong Ren (CAU). 1 female, China: Xinjiang, Burqin, Hemu, 48.43N, 87.57E, elev. 1040 m, 2016.VII.21, Jinlong Ren (CAU). 12 males, 9 females, China: Xinjiang, Burqin, Hemu, 48.58N, 87.45E, elev. 1160 m, 2016.VII.21, Jinlong Ren (CAU). 103 males, 58 females (CAU), China: Xinjiang, Burqin, Hemu, 48.56N, 87.44E, elev. 1150 m, 2016.VII.22, Jinlong Ren. 1 male, 1 female, China: Xinjiang, Burqin, Hemu, 48.572530N, 87.433929E, elev. 1090 m, 2016.VII.22, Jinlong Ren (light trap) (CAU). 276 males, 152 females, China: Xinjiang, Burqin, Hemu, 48.56N, 87.44E, elev. 1200 m, 2016.VII.23, Jinlong Ren (CAU).

#### Distribution.

China (Xinjiang: Burqin, Habahe), Japan (Hokkaido), Kazakhstan, Russia.

#### Remark.

This is the first report of this species from China.

### 7. Limonia taurica

Taxon classificationAnimaliaDipteraLimoniidae

(Strobl, 1895)

EC7BBCCD-DEAE-52E4-B2CF-4BF519A77F7E

Figures 55–57 , 58–61 , 62–63

Limnobia taurica Strobl 1895: 223.
Limonia sudetica Czizek 1931: 48 (synonymy).

#### Diagnosis.

Flagellar verticils 2.5 times longer than corresponding segment. Prescutum with five narrow, brown longitudinal stripes, except middle and lateral stripes dark brown. Wings with three dull-brown spots at basal Rs, apical Sc, and R_2_; Sc_1_ apically reaching 7/10 of Rs. Gonostylus with sharp apex and wider base. Apical paramere ended at 5/7 of aedeagus. Ventral margin of aedeagus with Y-shaped pattern.

#### Redescription.

Male (*n* = 3): body length 9–10 mm, wing length 7–8 mm.

**Figures 55–57.**
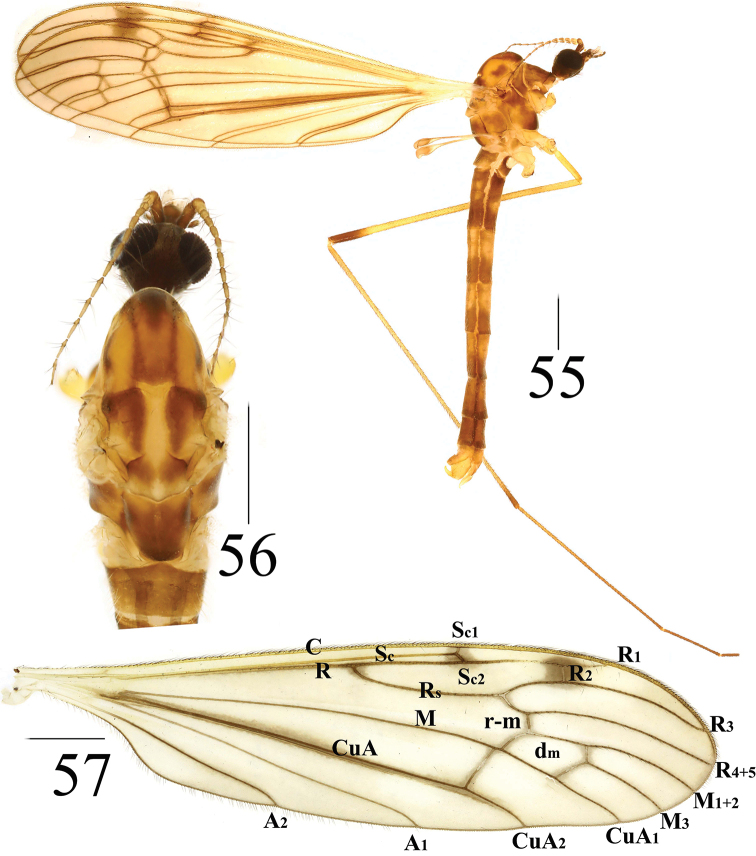
*Limonia taurica*, male **55** male habitus, lateral view **56** head and thorax, dorsal view **57** right wing. Scale bars: 1.0 mm.

***Head*** dark brown (Figs 55, 56). Vertex dark brown (Fig. 56). Occiput dull yellow, covered with black setulae. Antenna yellow and 14-segmented; scape and pedicel dull brown; flagellomeres brown, nearly cylindrical; flagellar verticils grayish yellow, 2.5 times longer than corresponding segment. Nasus 1/2 as long as rostrum. Labella pale yellow, covered with black setulae, except inner margin brown. Palpi brown, with black setulae.

***Thorax*** mostly brown (Figs 55, 56). Cervical sclerite dull brown, with black outer margin. Pronotum dark brown, with black setulae. Prescutum with five narrow, brown longitudinal stripes, except middle and lateral stripes dark brown, lateral margin of prescutum with oval, dark marking (Fig. 56). Scutum reddish brown, with triangular yellow marking at postero-lateral margin, covered with sparse setulae at anterior margin. Scutellum dull yellow, with pale yellow markings at posterior margin. Mediotergite reddish brown, with two dull yellow markings at posterior margin (Fig. 56). Mesopleura reddish brown; subspiracular sclerite reddish brown; katepisternum with black setulae. Legs with coxae and trochanters dull yellow, except frontal coxae brown; femora yellow with dark-brown subapical ring; tibiae yellow with reddish-brown subapical ring; tarsi yellow to dark brown onwards. Setulae on legs black. Wings brown with three dull-brown spots at basal Rs, apical Sc, and R_2_; Sc_1_ apically reaching 7/10 of Rs (Fig. 57). Halter: stem white to brown; knob brown but white at posterior part.

***Abdomen*** mainly pale brown (Fig. 55). Each abdominal segment with broad, reddish-brown ring at posterior margin. Abdominal segments 7 and 8 entirely reddish brown. Abdominal setulae golden.

**Figures 58–61.**
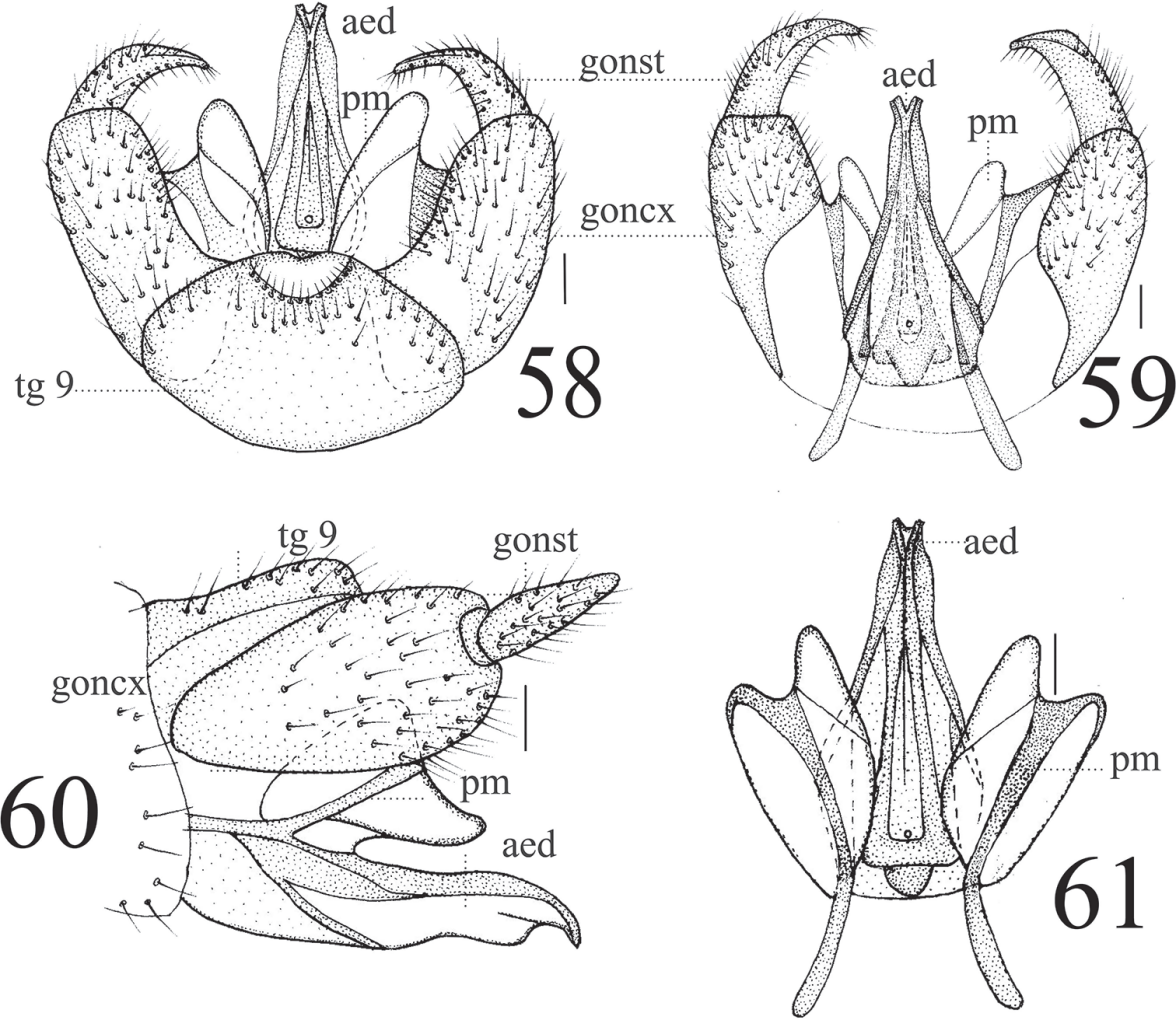
*Limonia taurica*, male **58** hypopygium, dorsal view **59** hypopygium, ventral view **60** hypopygium, lateral view **61** aedeagus and paramere. Scale bars: 0.1 mm.

***Hypopygium*** reddish brown (Figs 58–61). Posterior margin of tergite 9 slightly emarginated (Fig. 58). Gonocoxite long cylindrical. Gonostylus with sharp apex and the wider base (Figs 58, 59). Paramere with fan-shaped base and blunt apex, ending at 5/7 of aedeagus (Figs 58–61). Aedeagus forked; ventral margin with mid-protrusion triangular in lateral view; ventral margin with Y-shaped middle and anterior margin rounded (Figs 49, 51).

**Female** (*n* = 5): body length 8.5–9 mm, wing length 9–10 mm.

**Figures 62–63.**
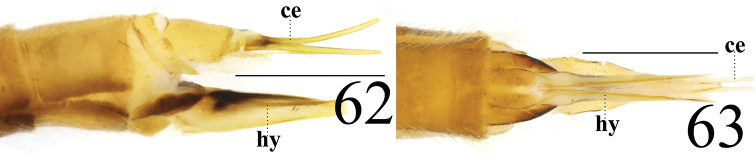
*Limonia taurica*, female. **62** cercus and hypogynial valves, lateral view **63** cercus and hypogynial valves, ventral view. Scale bars: 1.0 mm.

**Female** resembling male in head, thorax, and wing. Female terminalia pale yellow. Cercus yellow with slightly arched dorsally at apex, slender, and 3 times longer than wide at base. Hypogynial valve 5.3 times longer than wide at base; lateral margin with triangular, black marking (Figs 62, 63).

#### Material examined.

2 males, 2 females, China: Xinjiang, Habahe, Baihabacun, 48.69N, 86.79E, elev. 1170 m, 2016.VII.12, Bing Zhang (light trap) (CAU). 1 male, China: Xinjiang, Burqin, Kanas, 48.68N, 86.99E, elev. 1470 m, 2016.VII.16, Jinlong Ren (CAU). 2 females, China: Xinjiang, Habahe, Baihabacun, 48.69N, 86.78E, elev. 1170 m, 2016.VII.13, Bing Zhang (light trap) (CAU). 1 male, China: Xinjiang, Burqin, Hemu, 48.56N, 87.44E, elev. 1150 m, 2016.VII.22, Jinlong Ren (CAU). 1 female, China: Xinjiang, Burqin, Hemu, 48.56N, 87.44E, elev. 1200 m, 2016.VII.23, Jinlong Ren (CAU). 2 males, 1 female, China: Inner Mongolia, Hohehot, Xiaojinggou, 39.79N, 111.40E, elev. 1400 m, 2016.VII.27, Ding Yang (CAU).

#### Distribution.

Albania, Austria, Belgium, Bosnia-Herzegovina, Bulgaria, China (Xinjiang: Burqin, Habahe; Inner Mongolia: Hohehot), Czech Rep., France, Germany, Italy, Macedonia, Montenegro, Poland, Romania, Slovakia, Slovenia, Spain, Switzerland, Turkey, Ukraine.

#### Remark.

This is the first report of this species from China.

## Supplementary Material

XML Treatment for Limonia

XML Treatment for Limonia macrostigma

XML Treatment for Limonia medexocha

XML Treatment for Limonia phragmitidis

XML Treatment for Limonia stigma

XML Treatment for Limonia subcosta

XML Treatment for Limonia sylvicola

XML Treatment for Limonia taurica
